# Author Correction: Mechanisms governing the pioneering and redistribution capabilities of the non-classical pioneer PU.1

**DOI:** 10.1038/s41467-020-15012-6

**Published:** 2020-02-25

**Authors:** Julia Minderjahn, Andreas Schmidt, Andreas Fuchs, Rudolf Schill, Johanna Raithel, Magda Babina, Christian Schmidl, Claudia Gebhard, Sandra Schmidhofer, Karina Mendes, Anna Ratermann, Dagmar Glatz, Margit Nützel, Matthias Edinger, Petra Hoffmann, Rainer Spang, Gernot Längst, Axel Imhof, Michael Rehli

**Affiliations:** 1 Department of Internal Medicine III, University Hospital Regensburg, 93053 Regensburg, Germany; 2 Biomedical Center, Protein Analysis Unit, Faculty of Medicine, Ludwig-Maximilians-Universität München, Großhaderner Strasse 9, 82152 Planegg-Martinsried, Germany; 3 Biochemistry Centre Regensburg (BCR), University of Regensburg, 93053 Regensburg, Germany; 4 Statistical Bioinformatics Department, Institute of Functional Genomics, University of Regensburg, 93053 Regensburg, Germany; 5 Department of Dermatology and Allergy, Charité Universitätsmedizin Berlin, Berlin, Germany; 6 Regensburg Center for Interventional Immunology (RCI), University Regensburg and University Medical Center Regensburg, 93053 Regensburg, Germany; 7 Present Address: AstraZeneca, Tinsdaler Weg 183, 22880 Wedel, Germany; 8 Present Address: Rentschler Biopharma SE, 88471 Laupheim, Germany; 9 Present Address: Chromatin Structure and Cellular Senescence Research Unit, Maisonneuve-Rosemont Hospital Research Centre, Montréal, QC Canada H1T 2M4

**Keywords:** Gene regulation, Haematopoiesis, Chromatin remodelling, Nucleosomes, Transcription

Correction to: *Nature Communications* 10.1038/s41467-019-13960-2, published online 21 January 2020.

The original version of this Article contained an error in the spelling of the author Petra Hoffmann, which was incorrectly given as Petra Hoffman. This has been corrected in both the PDF and HTML versions of the Article.

