# Supplementary material for: Mechanisms governing the pioneering and redistribution capabilities of the non-classical pioneer PU.1
Source: Nat Commun. 2020 Jan 21;11:402. doi: 10.1038/s41467-019-13960-2 (PMC6972792; doi:10.1038/s41467-019-13960-2)
Supplement: Supplementary file 2 — Supplementary Information [file 41467_2019_13960_MOESM2_ESM.pdf]

Supplementary Information
for

Mechanisms governing the pioneering and redistribution capabilities of the non-classical pioneer PU.1

Julia Minderjahn,[1] Andreas Schmidt,[2] Andreas Fuchs,[3] Rudolf Schill,[4] Johanna Raithel,[1] Magda Babina,[5] Christian Schmidl,[6] Claudia Gebhard,[1,6] Sandra Schmidhofer,[1,7] Karina Mendes,[1] Anna Ratermann,[1,8] Dagmar Glatz,[1,9] Margit Nützel,[1] Matthias Edinger[1,6], Petra Hoffman[1,6], Rainer Spang,[4] Gernot Längst,[3] Axel Imhof,[2] Michael Rehli[1,6]

[1]Department of Internal Medicine III, University Hospital Regensburg, 93053 Regensburg, Germany

[2]Biomedical Center, Protein Analysis Unit, Faculty of Medicine, Ludwig-Maximilians-Universität München, Großhaderner Strasse 9, 82152 Planegg-Martinsried, Germany

[3]Biochemistry Centre Regensburg (BCR), University of Regensburg, 93053 Regensburg, Germany

[4]Statistical Bioinformatics Department, Institute of Functional Genomics, University of Regensburg, 93053 Regensburg, Germany

[5]Department of Dermatology and Allergy, Charité Universitätsmedizin Berlin, Berlin, Germany

[6]Regensburg Center for Interventional Immunology (RCI), University Regensburg and University Medical Center Regensburg, 93053 Regensburg, Germany

[7]present address: AstraZeneca, Tinsdaler Weg 183, 22880 Wedel, Deutschland

[8]present address: Rentschler Biopharma SE, 88471 Laupheim, Germany

[9]present address: Chromatin Structure and Cellular Senescence Research Unit, Maisonneuve-Rosemont Hospital Research Centre, Montréal, Quebec, Canada H1T 2M4

Supplement Index:

Supplementary Figures 1-6 page 02-09

Supplementary Tables 1-4 page 10-13

Supplementary References page 14

Supplementary Figures & Legends

Supplementary Figure 1

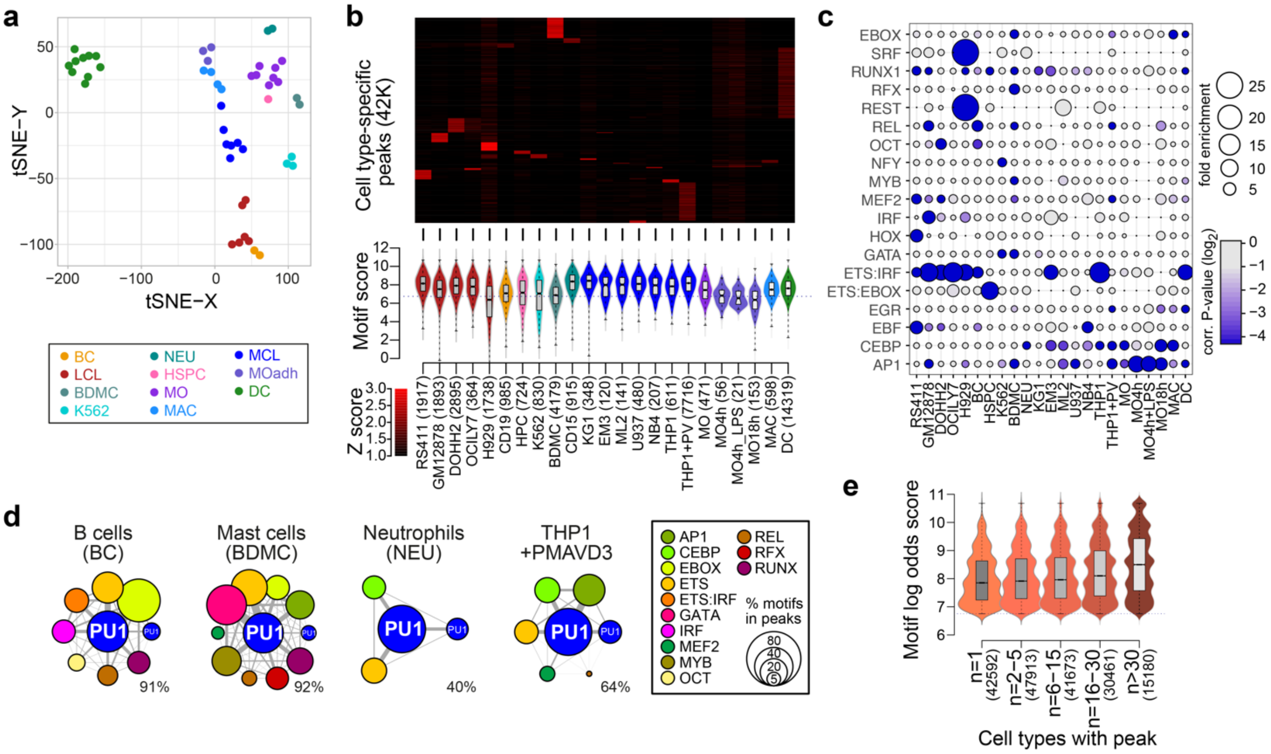

Cell type specific PU.1 binding, Related to Figure 1 (a) tSNE plot for individual cell types based on read count data of individual ChIP-seq experiments across the merged stringent peak set. Colors represent related cell types (LCL: lymphoid cell lines, MCL: myeloid cell lines, MOadh: adherent human blood monocytes) or biological replicates (BC: primary B cells, BDMC: breast skin-derived mast cells, NEU: primary neutrophils, HSPC: hematopoietic progenitor cells, MO: primary human blood monocytes, MAC: primary human MO-derived macrophages, DC: primary human MO-derived dendritic cells) (b) Cell type-specific PU.1 peaks and the frequency of their occurrence in the depicted cell type are shown in the heat map. The quantity of peaks in each cell type is given. The motif score distribution of the consensus MAC PU.1 motif, generated earlier, is shown for each cell type. The median of the specific distribution across all clusters is depicted inside the bean plot with a conventional boxplot. (c) The balloon plot depicts the motif enrichment of the selected motifs in each cell type. The node thickness represents the fold enrichment compared to random genomic background and the coloring correlates with the p-value of the motif co-occurrence in PU.1 peaks of each cell type. (d) Motif co-enrichment networks for four selected cell types. The fraction of PU.1 peaks overlapping with co-associated TF motifs other than PU.1 (in blue) is shown below each network. The size of each node represents the relative motif enrichment and the coloring indicates the co-associated TF motif. (e) Distribution of motif scores across cell type-specific peaks (n=1), peaks observed in several cell type (n ≤ 30) or more common peaks (n > 30). (a-e) Source data are provided as a Source Data file.

Supplementary Figure 2

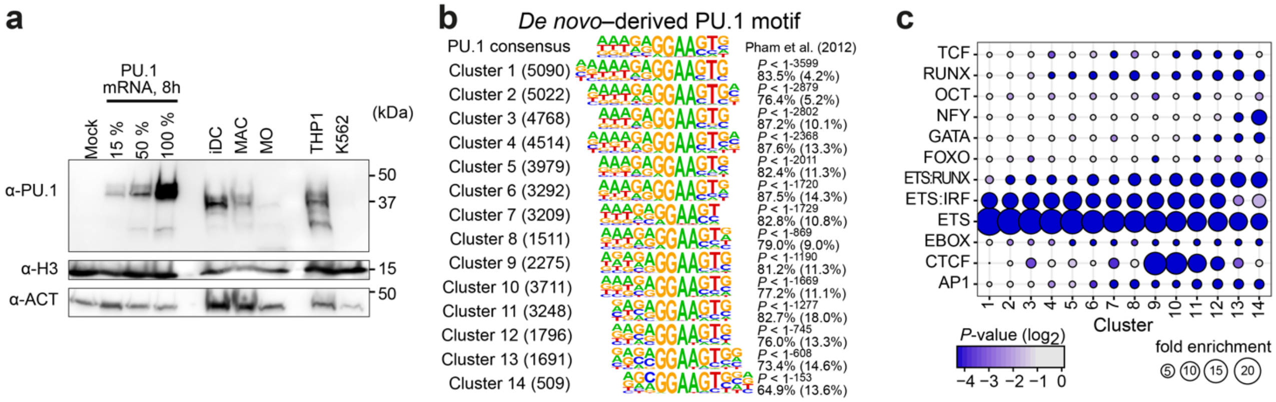

PU.1 expression and clusters of PU.1 peaks, Related to Figures 2 and 3 (a) Immunoblotting of whole cell extracts from mRNA transfected CTV-1 cells (8h after electroporation) as well as from monocyte-derived dendritic cells (iDC), monocyte-derived macrophages, monocytes, and two myeloid cell lines (THP-1, K562) using the indicated antibodies. (b) *De novo*-derived PU.1 motif enrichment across ATAC-seq clusters. Enrichment P values (hypergeometric test) as well as the fraction of motifs in peaks or in background regions (in brackets) are given. (c) Balloon plot depicting the motif enrichment of the selected motifs in each PU.1 peak cluster. The balloon size represents the fold-enrichment and the coloring indicates the corrected P-value (Hypergeometric test, Benjamini-Hochberg multiple testing correction) of the motif occurrence in PU.1 peaks across K-means clusters. (a-c) Source data are provided as a Source Data file.

Supplementary Figure 3

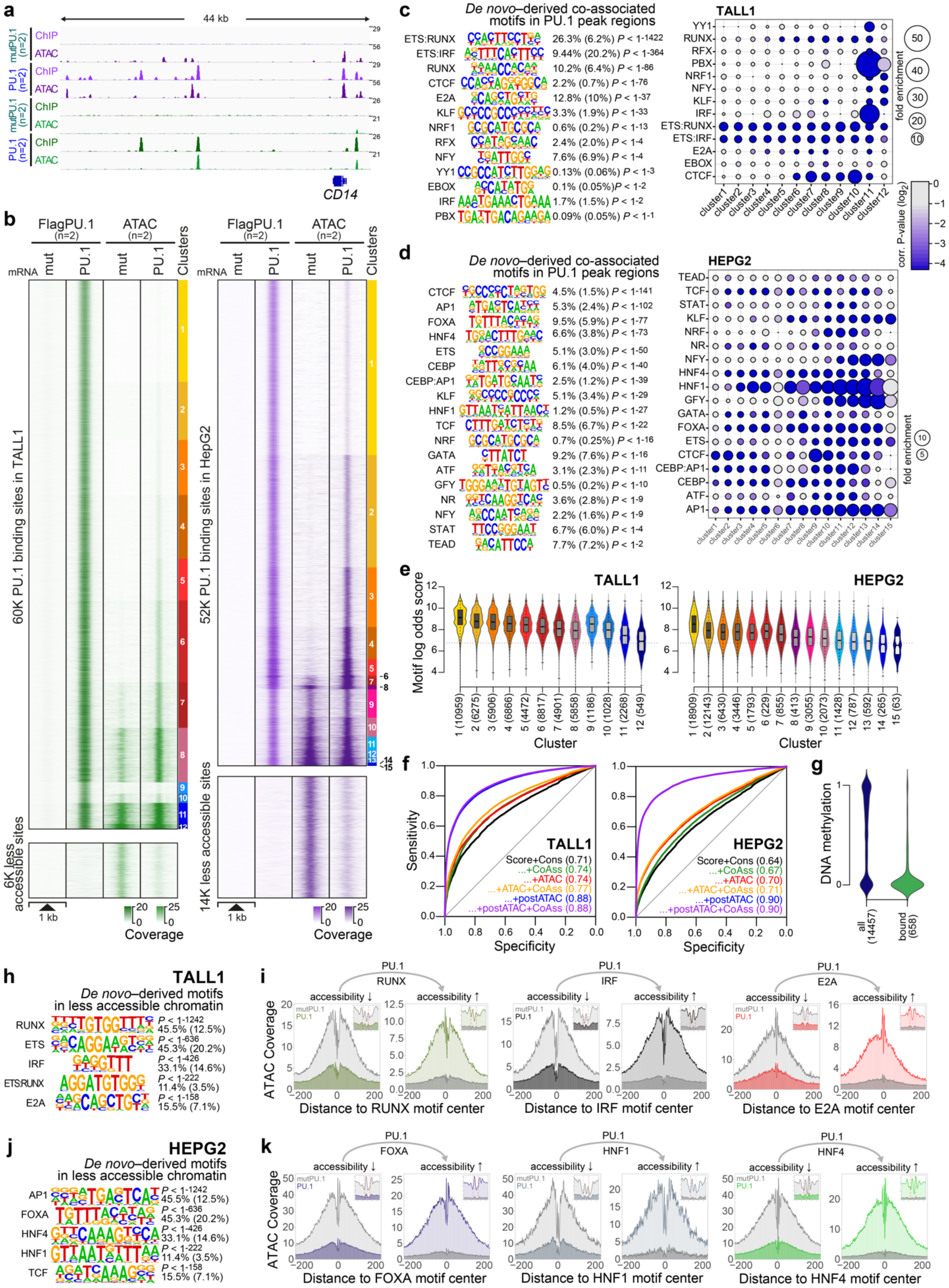

PU.1 induced chromatin remodeling in the two cell lines TALL1 and HepG2, Related to Figure 3, 4 and 5 (a) IGV genome browser tracks showing PU.1 ChIP-seq as well as ATAC-seq coverage in control (mutPU.1) and PU.1 expressing TALL1 (green tracks) and HepG2 cells (purple tracks) across an example locus. (b) Distribution of PU.1 ChIP-seq and ATAC-seq signals of PU.1- vs. PU.1mut transfected cells are plotted across 1 kb windows and all PU.1 binding sites (top panels) or regions that lost accessibility after PU.1 induction (bottom panels) in TALL1 (green plot) and HepG2 (purple plot). PU.1 peaks are ordered according to K-means clustering of peak-centered ATAC-seq signals in each cell type. PU.1 peak clusters are indicated by the color bar on the right. (c,d) *De novo*-derived co-associated motifs enriched across ATAC-seq-based PU.1 peak clusters and their enrichment across PU.1 peak clusters (depicted as balloon plots) are shown for TALL1 (in c) and HepG2 (in d) as described in Supplementary Figure 2b,c. (e) PU.1 motif log odds score distribution of the consensus PU.1 motif is shown for both cell lines as described in Figure 4a. (f) ROC curves for PU.1 binding prediction in TALL1 and HepG2 from logistic models as described in Figure 4f. (g) Distribution of DNA methylation ratios in HepG2 cells across PU.1 motifs containing a CpG next to the GGAA core recognition sequence, where binding was sensitive to DNA methylation. Distributions are shown for all single motifs (blue) and the subgroup of motifs bound in HepG2 (green). (h,j) *De novo*-derived motifs enriched across regions that lost accessibility upon PU.1 induction TALL1 (in h) and HepG2 (in j) as described in Figure 5a. (i,k). Representative ATAC-seq footprints across enriched motif-centered peaks that either lost or gained accessibility upon PU.1 induction in TALL1 (in i) or HepG2 (in k). Footprints of control cells (mutPU.1) are in grey, footprints of PU.1 mRNA transfected cells are colored. Smaller histograms in the upper right corner zoom into the central part of the main graph. The position of each motif is indicated in each inlay by two vertical dashed lines. (b-k) Source data are provided as a Source Data file.

Supplementary Figure 4

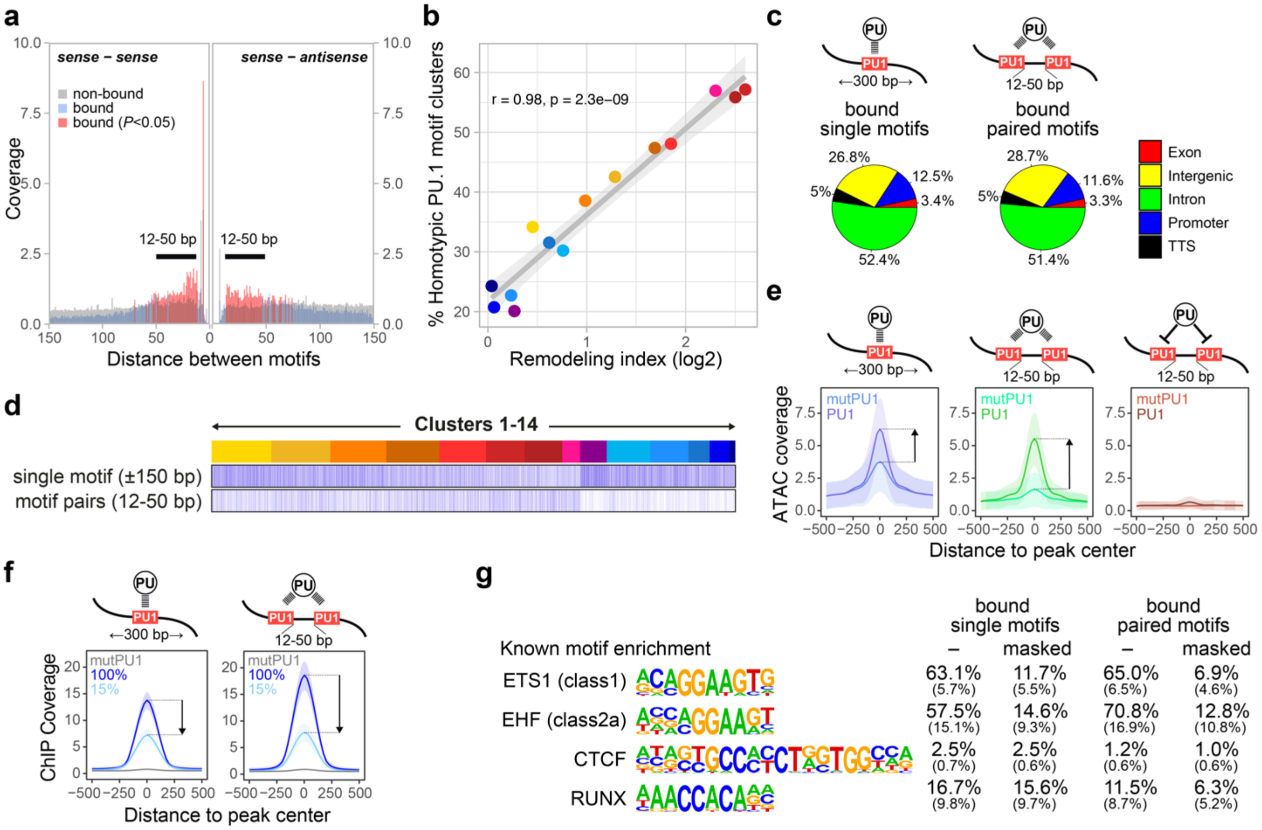

PU.1 motif pairs, Related to Figures 4 and 6 (a) The coverage of PU.1 motifs is plotted against the distance between PU.1 motifs across PU.1-bound vs. non-bound ChIP-seq binding sites. Significantly enriched PU.1-bound regions occurring between PU.1 motifs are depicted in orange (*P* < 0.05, hypergeometric test), whereas the overall coverage of PU.1-bound regions is depicted in blue vs. non-bound in grey. (b) Scatter plot depicting the Pearsons'correlation (significance calculated based on the alternative hypothesis that the true correlation is not equal to 0), between homotypic PU.1 motif clusters and the PU.1-induced Remodeling index. (c) Genome ontology analyses of PU.1-bound single vs. paired motifs. (d) Enrichment of PU.1-bound single vs. paired motifs across ATAC-seq-derived K-means clusters. (e) Histogram plots illustrating the ATAC-seq coverage of PU.1 vs. mutPU.1 in bound and unbound regions containing single and clustered PU.1 motifs (from left to right: PU.1 (purple) vs. mutPU.1 (blue) across single binding sites; PU.1 (green) vs. mutPU.1 (turquoise) coverage across clustered binding sites; PU.1 (brown) vs. mutPU.1 (light-brown) coverage across unbound clustered binding sites. Shaded areas mark the 95% confidence interval of the average coverages. (f) Histogram plots illustrating the PU.1 ChIP-seq coverage across single vs. clustered binding sites for different PU.1 titration levels. Shaded areas mark the 95% confidence interval of the average coverages. (g) Known motif enrichment analyses across PU.1-bound single vs. paired motifs including the PU.1 motif or with the PU.1 motif masked. (a-g) Source data are provided as a Source Data file.

Supplementary Figure 5

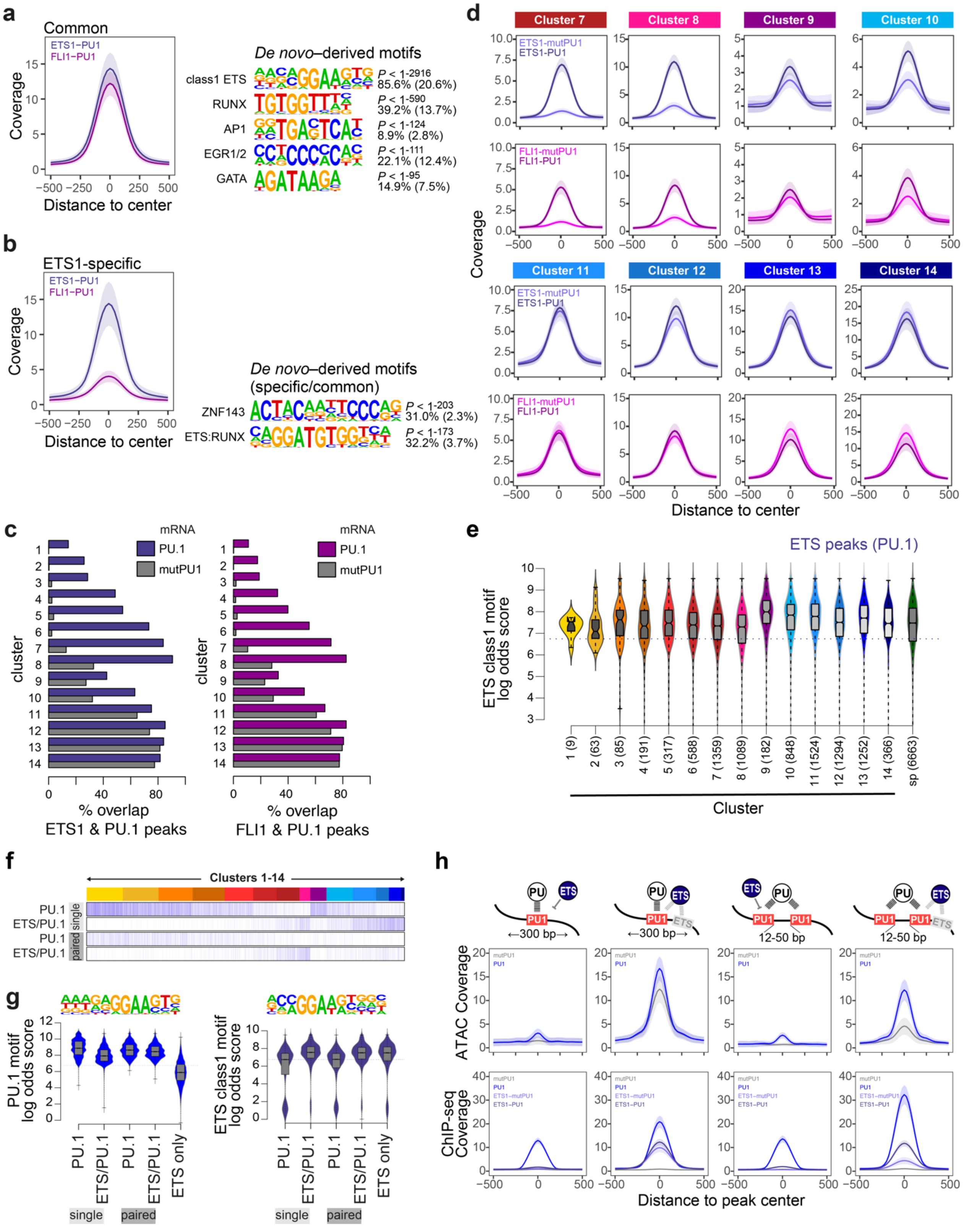

ETS1 and FLI1 overlap with PU.1, Related to Figure 4 (a,b) Enrichment of ETS1 and FLI1 ChIP-seq reads (histogram left panel) and *de novo*-derived motif co-enrichment across common and ETS1-specific ChIP-seq peaks. Shaded areas in histograms mark the 95% confidence interval of the average coverages. Enrichment P values (hypergeometric test) as well as the fraction of motifs in peaks or in background regions (in brackets) are given for each co-enriched motif. (c) Bar plots displaying the overlap of standard ETS1 (left panel) and FLI1 (right panel) peaks in PU.1-transfected (blue/purple bars) and control CTV-1 cells not expressing PU.1 (grey bars) with PU.1 peaks across the 14 ATAC-seq-derived K-means clusters introduced in Fig. 3. (d) ETS1 and FLI1 ChIP-seq coverage across PU.1 peaks in clusters 7-14. (e) Motif log odds score distribution of the consensus ETS class 1 motif is shown for ETS1-overlapping peaks across ATAC-seq-derived PU.1 peak clusters along with specific (sp) peaks of ETS1. The median of each distribution is depicted inside the bean with a conventional boxplot. (f) Enrichment of PU.1-bound single vs. paired motifs either bound by PU1 alone, or together with ETS factors (ETS/PU.1) across ATAC-seq-derived K-means clusters. (g) Motif log odds score distributions of the consensus PU.1 and ETS class 1 motifs are shown for PU.1-bound single vs. paired motifs either bound by PU.1 alone, or together with ETS-factors (ETS/PU.1) in comparison with ETS-specific (only) peaks. The median of each distribution is depicted inside the bean with a conventional boxplot. (h) Histogram plots illustrating the ATAC-seq coverage as well as the ChIP-seq coverage of PU.1 (blue) and ETS1 (dark-purple) in PU.1 mRNA-transfected cells, or PU.1 (grey), and ETS1 (light-purple) in PU.1mut CTV-1 cells across single vs. paired motifs either bound by PU.1 alone, or together with ETS-factors (ETS/PU.1). (a-h) Source data are provided as a Source Data file.

Supplementary Figure 6

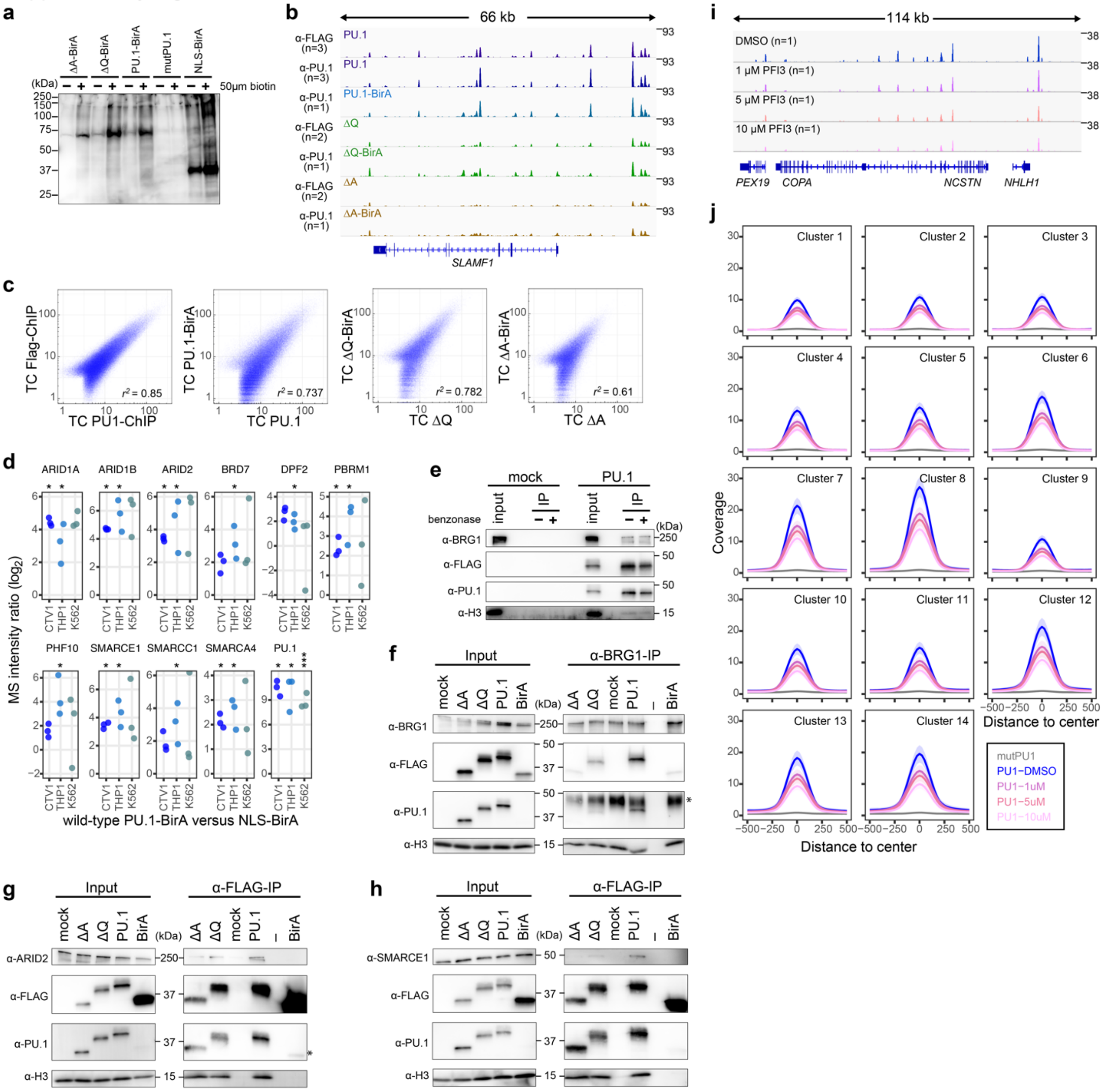

Identification of PU.1 proximal proteins using BioID and effect of BRG inhibition, Related to Figure 8 (a) Western blot for biotin-labelled proteins in whole cell lyses from CTV-1 cells transfected with mRNAs for the indicated BirA-fusion proteins or control mRNA and cultured in the presence or absence of biotin. (b) IGV genome browser track of the *SLAMF6* locus showing αPU.1 or αFLAG ChIP-seq coverage of CTV-1 cells transfected with the indicated mRNAs either fused to *E.coli* biotin ligase (BirA) or not. (c) Scatter plots comparing PU.1 read counts of CTV-1 cells transfected with various fusion proteins. The Pearson correlation coefficient is given in the lower right corner of each plot. (d) Dot plots showing the enrichment of peptides representing the indicated SWI/SNF components (PU.1 is shown as a control) in BioIDs from PU.1-BirA versus NLS-BirA mRNA transfected cells lines (***, $P<0.001$; **, $P<0.01$; *, $P<0.05$; paired t-test, permutation-based correction). (e) Immunoblotting of αFLAG mAb immunoprecipitations (5h after electroporation, IP 5h) treated or not with benzonase, along with corresponding input lysates of control (mock) and PU.1 mRNA transfected cells using the indicated antibodies. (F-H) Immunoblotting of αBRG1 (f) or αFLAG (g,h) mAb immunoprecipitations (5h after electroporation, IP 5h), along with corresponding input lysates of control (mock), PU.1, ΔQ, ΔA and BirA mRNA transfected cells using the indicated antibodies. Asterisks mark unspecific bands. (i) IGV genome browser track of an exemplary locus showing PU.1 ChIP-seq coverage of PU.1-transfected CTV-1 cells treated with varying amounts of the small molecule inhibitor PFI-3 or a DMSO control. (j) ChIP-seq coverage of PU.1-transfected CTV-1 cells treated with varying amounts of PFI-3 across PU.1 peaks in clusters 1–14. Control cells treated with DMSO (blue) or transfected with PU.1mut mRNA (grey) are shown in comparison. Shaded areas mark the 95% confidence interval of the average coverages. (a,c-h,j) Source data are provided as a Source Data file.

Supplementary Table 1
Summary of published NGS data used in this study

ChIP-sequencing

Cell type	Epitope	SRA acc.	Total reads[1]	FRIP[2] (%)	Standard peaks	Stringent peaks	Reference
DOHH2	PU.1	SRR2050990	32908148	27.02	69630	31136	[1]
	Input	SRR2050987	27088253	–	–	–	
GM12878	PU.1	SRR351880,81 SRR578180,81	70486938	19.24	66120	24227	[2]
	Input	SRR351535	34799129	–	–	–	
H929	PU.1	SRR1240634	23323988	9.42	25369	10640	[3]
	Input	SRR1240632	26905897	–	–	–	
K562	PU.1	SRR351605	14123551	6.44	25329	9432	[2]
	Input	SRR351541	22208974	–	–	–	
	PU.1	SRR2085865	12780053	4.24	15456	8284	[4]
	Input	SRR2085862	29198647	–	–	–	
	PU.1	SRR2085866	12659155	15.19	42948	25938	[4]
	Input	SRR2085863	11474429	–	–	–	
OCILY7	PU.1	SRR2051012	26041338	10.89	36977	14667	[1]
	Input	SRR2051009	33425026	–	–	–	
RS411 untreated	PU.1	SRR2138399	10564818	7.26	13090	13090	[5]
	Input	SRR2138398	3141697	–	–	–	
RS411 Dex 1h	PU.1	SRR2138409	10933065	12.77	21554	21376	[5]
	Input	SRR2138408	3298293	–	–	–	
Human hematopoietic stem & progenitor cells (HSPC)	PU.1	SRR094808,9	9467756	8.84	21064	15124	[6]
	Input	SRR094805	6259718	–	–	–	
Human monocytes (MO)	PU.1	SRR333627-32	16782798	19.16	68674	36210	[7]
Human macrophages (MAC)	PU.1	SRR333643-8	16274812	21.89	87057	43884	[7]
	Input	SRR333634-6	13796175	–	–	–	
MO subsets: Input DonorA	Input	SRR548292	30643280	–	–	–	[8]
MO subsets: Input DonorD	Input	SRR548293	32343363	–	–	–	[8]
B cells: Input DonorA	Input	SRR557727	27712464	–	–	–	[9]
B cells: Input DonorC	Input	SRR557730	27569098	–	–	–	[9]

[1]Unique reads after mapping to human reference genome hg19
[2]Fraction of reads in peaks (FRIP), determined by running HOMER's findPeaks program in ''factor'' mode using default parameters and the matching background (input) [10]
[3]Number of peaks (determined by HOMER's findPeaks program in ''factor'' mode on CNV-normalized data and filtering for mappability)
[4]Number of peaks (determined by HOMER's findPeaks program in ''factor'' mode on CNV-normalized data, -fdr 0.00001, minimum of $15/10^7$ reads, and filtering for mappability)

WGBS-sequencing

Cell type	GEO acc.	Data	Reference
HepG2	GSM1204463	Bed file containing all seen CpGs as provided	[11]

Supplementary Table 2

Gene blocks nucleosome assemblies

Construct name	gBlock sequences (5'--3')[1]
No_motif	AATTCGGATCCCACGATAAGCATAACCAAGCTCTGCGATTATCTCTACCATAATTAATTTAAGCAGCC<br>GTATTTATAAAGAAATTTCCAAAATAAAGCGAATATTCTAGAATCCCAAAACAAACTGGTTGTTGCGG<br>TAGGTCATTTGTTTGGCAGATCCAGAATCCTGGTGCTGAGGCTGCTCAATTGGTTGTAGCAAGCTCTA<br>GCACTGCTTAAATGCATGTACGCGCGGTCCCCTGTGTTTTAACTGCCAAGGGGATTACTCCCTAGTCT<br>CCAGGCATGTGTCAGATATATACAGCTAATCAGTTCTCCGGGTTGTCAGGTCGACCAGTTGTTCCTTT<br>GAGGTCCGGTTCTTTTCGTTATGGGGTCATTTTTGGGCCACCTCCCCAGGTATGACTTCCAGGTATTC<br>TCTGTGGCCTGTCACTTTCCTCCCTGTCTCTTTTATGCTTGTGATCTTTTCTATCTGTTCCTATTGGA<br>CCTGGAGATAGGTACTGACACGCT
Dyad_axis	AATTCGGATCCCACGATAAGCATAACCAAGCTCTGCGATTATCTCTACCATAATTAATTTAAGCAGCC<br>GTATTTATAAAGAAATTTCCAAAATAAAGCGAATATTCTAGAATCCCAAAACAAACTGGTTGTTGCGG<br>TAGGTCATTTGTTTGGCAGATCCAGAATCCTGGTGCTGAGGCTGCTCAATTGGTTGTAGCAAGCTCTA<br>GCACTGCTTAAATGCAT**CACTTCCTCTTT**CCCTGTGTTTTAACTGCCAAGGGGATTACTCCCTAGTCT<br>CCAGGCATGTGTCAGATATATACAGCTAATCAGTTCTCCGGGTTGTCAGGTCGACCAGTTGTTCCTTT<br>GAGGTCCGGTTCTTTTCGTTATGGGGTCATTTTTGGGCCACCTCCCCAGGTATGACTTCCAGGTATTC<br>TCTGTGGCCTGTCACTTTCCTCCCTGTCTCTTTTATGCTTGTGATCTTTTCTATCTGTTCCTATTGGA<br>CCTGGAGATAGGTACTGACACGCT
Motif_13-bp_inside	AATTCGGATCCCACGATAAGCATAACCAAGCTCTGCGATTATCTCTACCATAATTAATTTAAGCAGCC<br>GTATTTATAAAGAAATTTCCAAAATAAAGCGAATATTCTAGAATCCCAAAACAAACTGGTTGTTGCGG<br>TAGGTCATTTGTTTGGCAGATCCAGAATCCTGGTGCTGAGGCTGCTCAATTGGTTGTAGCAAGCTCTA<br>GCACTGCTTAAATGCATGTACGCGCGGTCCCCTGTGTTTTAACTGCCAAGGGGATTACTCCCTAGTCT<br>CCA**CACTTCCTCTTT**ATATATACAGCTAATCAGTTCTCCGGGTTGTCAGGTCGACCAGTTGTTCCTTT<br>GAGGTCCGGTTCTTTTCGTTATGGGGTCATTTTTGGGCCACCTCCCCAGGTATGACTTCCAGGTATTC<br>TCTGTGGCCTGTCACTTTCCTCCCTGTCTCTTTTATGCTTGTGATCTTTTCTATCTGTTCCTATTGGA<br>CCTGGAGATAGGTACTGACACGCT
Motif_10-bp_inside	AATTCGGATCCCACGATAAGCATAACCAAGCTCTGCGATTATCTCTACCATAATTAATTTAAGCAGCC<br>GTATTTATAAAGAAATTTCCAAAATAAAGCGAATATTCTAGAATCCCAAAACAAACTGGTTGTTGCGG<br>TAGGTCATTTGTTTGGCAGATCCAGAATCCTGGTGCTGAGGCTGCTCAATTGGTTGTAGCAAGCTCTA<br>GCACTGCTTAAATGCATGTACGCGCGGTCCCCTGTGTTTTAACTGCCAAGGGGATTACTCCCTAGTCT<br>CCAGGC**CACTTCCTCTTT**TATACAGCTAATCAGTTCTCCGGGTTGTCAGGTCGACCAGTTGTTCCTTT<br>GAGGTCCGGTTCTTTTCGTTATGGGGTCATTTTTGGGCCACCTCCCCAGGTATGACTTCCAGGTATTC<br>TCTGTGGCCTGTCACTTTCCTCCCTGTCTCTTTTATGCTTGTGATCTTTTCTATCTGTTCCTATTGGA<br>CCTGGAGATAGGTACTGACACGCT
Motif_10-bp_outside	AATTCGGATCCCACGATAAGCATAACCAAGCTCTGCGATTATCTCTACCATAATTAATTTAAGCAGCC<br>GTATTTATAAAGAAATTTCCAAAATAAAGCGAATATTCTAGAATCCCAAAACAAACTGGTTGTTGCGG<br>TAGGTCATTTGTTTGGCAGATCCAGAATCCTGGTGCTGAGGCTGCTCAATTGGTTGTAGCAAGCTCTA<br>GCACTGCTTAAATGCATGTACGCGCGGTCCCCTGTGTTTTAACTGCCAAGGGGATTACTCCCTAGTCT<br>CCAGGCATGTGTCAGATATATACAGCTAATCAGTT**CACTTCCTCTTT**AGGTCGACCAGTTGTTCCTTT<br>GAGGTCCGGTTCTTTTCGTTATGGGGTCATTTTTGGGCCACCTCCCCAGGTATGACTTCCAGGTATTC<br>TCTGTGGCCTGTCACTTTCCTCCCTGTCTCTTTTATGCTTGTGATCTTTTCTATCTGTTCCTATTGGA<br>CCTGGAGATAGGTACTGACACGCT

[1]The dyad axis is marked in gray, the high affinity PU.1 sequence in red lettering

Supplementary Table 3
Oligonucleotides

Primer name	Primer sequence (5‘--3‘)	Experiment
NPS-forward[1]	GATCCAGAATCCTGGTGCT	EMSA
NPS-reverse[2]	AAAGGAACAACTGGTCGACC	EMSA
5C_distal_sense[3]	acgtAAAGAGGAAGCGacgt	MST
5mC_distal_sense	acgtAAAGAGGAAG(5mC)Gacgt	MST
5hmC_distal_sense	acgtAAAGAGGAAG(5hmC)Gacgt	MST
5C_proximal_sense	acgtGAAGCGGAAGTGacgt	MST
5mC_proximal_sense	acgtGAAG(5mC)GGAAGTGacgt	MST
5hmC_proximal_sense	acgtGAAG(5hmC)GGAAGTGacgt	MST

[1]For amplification of NPS from gene blocks used with a 5’ Cy3 and Cy5 label
[2]For amplification of NPS with 40 bp overhang on 3’ side
[3]Only sense strand is shown

Supplementary Table 4

Summary of BioID proteomics data generated in this study

(ProteomeXchange dataset identifier: PXD013167)

Sample	Transfected mRNA	Batch
CTV-1	PU.1-BirA	1
	PU.1-BirA	2
	PU.1-BirA	3
CTV-1	PU.1-ΔQ-BirA	1
	PU.1-ΔQ-BirA	2
	PU.1-ΔQ-BirA	3
CTV-1	PU.1-ΔA-BirA	1
	PU.1-ΔA-BirA	2
	PU.1-ΔA-BirA	3
CTV-1	NLS-BirA	1
	NLS-BirA	2
	NLS-BirA	3
THP-1	PU.1-BirA	1
	PU.1-BirA	2
	PU.1-BirA	3
THP1	NLS-BirA	1
	NLS-BirA	2
	NLS-BirA	3
K562	PU.1-BirA	1
	PU.1-BirA	2
	PU.1-BirA	3
K562	NLS-BirA	1
	NLS-BirA	2
	NLS-BirA	3

Supplementary References

1. Ryan RJH, *et al.* Detection of Enhancer-Associated Rearrangements Reveals Mechanisms of Oncogene Dysregulation in B-cell Lymphoma. *Cancer Discovery* **5**, 1058-1071 (2015).

2. Gertz J, *et al.* Distinct Properties of Cell-Type-Specific and Shared Transcription Factor Binding Sites. *Molecular Cell* **52**, 25-36 (2013).

3. Care MA, *et al.* SPIB and BATF provide alternate determinants of IRF4 occupancy in diffuse large B-cell lymphoma linked to disease heterogeneity. *Nucleic Acids Research* **42**, 7588-7610 (2014).

4. Schmidl C, Rendeiro AF, Sheffield NC, Bock C. ChIPmentation: fast, robust, low-input ChIP-seq for histones and transcription factors. *Nat Methods* **12**, 963-965 (2015).

5. Wu JN, Pinello L, Yissachar E, Wischhusen JW, Yuan GC, Roberts CWM. Functionally distinct patterns of nucleosome remodeling at enhancers in glucocorticoid-treated acute lymphoblastic leukemia. *Epigenetics & Chromatin* **8**, (2015).

6. Cui K, *et al.* Chromatin signatures in multipotent human hematopoietic stem cells indicate the fate of bivalent genes during differentiation. *Cell Stem Cell* **4**, 80-93 (2009).

7. Pham TH, *et al.* Dynamic epigenetic enhancer signatures reveal key transcription factors associated with monocytic differentiation states. *Blood* **119**, e161-171 (2012).

8. Schmidl C, *et al.* Transcription and enhancer profiling in human monocyte subsets. *Blood* **123**, e90-99 (2014).

9. Andersson R, *et al.* An atlas of active enhancers across human cell types and tissues. *Nature* **507**, 455-461 (2014).

10. Heinz S, *et al.* Simple combinations of lineage-determining transcription factors prime cis-regulatory elements required for macrophage and B cell identities. *Mol Cell* **38**, 576-589 (2010).

11. Ziller MJ, *et al.* Charting a dynamic DNA methylation landscape of the human genome. *Nature* **500**, 477-481 (2013).
