# Supplementary material for: Mechanisms governing the pioneering and redistribution capabilities of the non-classical pioneer PU.1
Source: Nat Commun. 2020 Jan 21;11:402. doi: 10.1038/s41467-019-13960-2 (PMC6972792; doi:10.1038/s41467-019-13960-2)
Supplement: Supplementary file 7 — Source data [file 41467_2019_13960_MOESM7_ESM.zip › Source_Data/Figure5/Figure5A_MotifScanOutput/homerResults.html]

/misc/data/analysis/project\_PU1/CTV1/motifs/basic/ATAC\_PU1mutvsPU1.peaks.DESEQ/final - Homer de novo Motif Results


# Homer *de novo* Motif Results (/misc/data/analysis/project\_PU1/CTV1/motifs/basic/ATAC\_PU1mutvsPU1.peaks.DESEQ/final)

Known Motif Enrichment Results  
Gene Ontology Enrichment Results  
If Homer is having trouble matching a motif to a known motif, try copy/pasting the matrix file into
STAMP  
More information on motif finding results: HOMER
| Description of Results
| Tips
  
Total target sequences = 3016  
Total background sequences = 45623  
\* - possible false positive  

|  |  |  |  |  |  |  |  |  |
| --- | --- | --- | --- | --- | --- | --- | --- | --- |
| Rank | Motif | P-value | log P-pvalue | % of Targets | % of Background | STD(Bg STD) | Best Match/Details | Motif File |
| 1 | C A G T A C G T G A C T C A G T A C T G G C A T C A T G A T C G A G C T G A C T C G A T | 1e-455 | -1.049e+03 | 52.39% | 15.00% | 148.6bp (138.1bp) | RUNX1(Runt)/Jurkat-RUNX1-ChIP-Seq(GSE29180)/Homer(0.994) More Information | Similar Motifs Found | motif file (matrix) |
| 2 | T G A C G A T C C T G A A G T C G C A T A G C T G T A C A G T C C G A T A C T G A G C T T A G C | 1e-181 | -4.170e+02 | 26.56% | 8.10% | 175.2bp (134.4bp) | ERG(ETS)/VCaP-ERG-ChIP-Seq(GSE14097)/Homer(0.940) More Information | Similar Motifs Found | motif file (matrix) |
| 3 | T A G C G C T A A T G C G C T A C G A T T A G C C G T A T G C A C G T A T A C G T A C G | 1e-146 | -3.382e+02 | 43.14% | 21.30% | 163.9bp (136.7bp) | TCF7L2/MA0523.1/Jaspar(0.951) More Information | Similar Motifs Found | motif file (matrix) |
| 4 | A C T G A G T C G A C T C G A T C G T A C A G T A T G C C G A T | 1e-129 | -2.990e+02 | 35.25% | 16.28% | 152.9bp (135.1bp) | GATA3/MA0037.2/Jaspar(0.975) More Information | Similar Motifs Found | motif file (matrix) |
| 5 | T C G A C G A T A T C G C G T A T A G C C G A T T G A C C G T A A C G T G T C A A G C T | 1e-116 | -2.674e+02 | 18.73% | 5.95% | 141.2bp (136.9bp) | BATF(bZIP)/Th17-BATF-ChIP-Seq(GSE39756)/Homer(0.990) More Information | Similar Motifs Found | motif file (matrix) |
| 6 | G A T C C T A G G T A C C G A T A C T G C G A T C T A G T C A G A G C T T C G A T C G A | 1e-81 | -1.887e+02 | 26.26% | 12.69% | 168.4bp (137.6bp) | RUNX-AML(Runt)/CD4+-PolII-ChIP-Seq(Barski\_et\_al.)/Homer(0.823) More Information | Similar Motifs Found | motif file (matrix) |
| 7 | A T G C A G T C T C G A A G T C T C G A A T C G G C T A | 1e-76 | -1.759e+02 | 49.37% | 32.45% | 212.7bp (139.8bp) | POL009.1\_DCE\_S\_II/Jaspar(0.786) More Information | Similar Motifs Found | motif file (matrix) |
| 8 | A T G C C T A G G T A C G T A C C T G A A T G C T G C A C T A G | 1e-65 | -1.512e+02 | 16.71% | 7.01% | 176.0bp (146.2bp) | RUNX2(Runt)/PCa-RUNX2-ChIP-Seq(GSE33889)/Homer(0.732) More Information | Similar Motifs Found | motif file (matrix) |
| 9 | C G T A C G T A A G T C A G T C C T A G A G T C A G T C | 1e-62 | -1.433e+02 | 26.06% | 14.02% | 170.1bp (137.0bp) | MYB(HTH)/ERMYB-Myb-ChIPSeq(GSE22095)/Homer(0.883) More Information | Similar Motifs Found | motif file (matrix) |
| 10 | G T C A T C G A A G T C A G T C A G T C G T C A A G T C C T A G | 1e-59 | -1.361e+02 | 24.34% | 12.95% | 181.0bp (140.8bp) | Egr2(Zf)/Thymocytes-Egr2-ChIP-Seq(GSE34254)/Homer(0.854) More Information | Similar Motifs Found | motif file (matrix) |
| 11 | G T A C G T A C C T A G G A T C G C T A G A C T A G T C A T G C C A T G A C G T | 1e-56 | -1.304e+02 | 7.03% | 1.73% | 119.6bp (126.0bp) | ETS:RUNX(ETS,Runt)/Jurkat-RUNX1-ChIP-Seq(GSE17954)/Homer(0.776) More Information | Similar Motifs Found | motif file (matrix) |
| 12 | A G T C C A G T A C T G A C G T C T G A C T A G A C T G A G T C A C G T A G T C A G T C C T G A A G T C A G T C | 1e-55 | -1.288e+02 | 2.88% | 0.20% | 96.6bp (138.7bp) | ZNF692(Zf)/HEK293-ZNF692.GFP-ChIP-Seq(GSE58341)/Homer(0.672) More Information | Similar Motifs Found | motif file (matrix) |
| 13 | A C T G A C T G A G T C C G A T A C T G A G T C A C G T A C G T A C G T A C T G A C G T A C G T A C G T C G T A | 1e-54 | -1.247e+02 | 2.95% | 0.23% | 96.6bp (136.6bp) | FOXP1/MA0481.1/Jaspar(0.702) More Information | Similar Motifs Found | motif file (matrix) |
| 14 | G C T A G C T A A G C T T G A C C G T A T G A C C G T A A C T G G T A C C G A T A C T G G A C T A C G T G C T A | 1e-53 | -1.224e+02 | 23.94% | 13.15% | 187.0bp (142.4bp) | Myf5(bHLH)/GM-Myf5-ChIP-Seq(GSE24852)/Homer(0.851) More Information | Similar Motifs Found | motif file (matrix) |
| 15 | A C T G A G T C C G A T T A G C A G T C C G T A A G T C A G T C T G A C G T C A A T C G C A G T C G A T | 1e-52 | -1.201e+02 | 2.88% | 0.23% | 87.4bp (125.6bp) | Sp5(Zf)/mES-Sp5.Flag-ChIP-Seq(GSE72989)/Homer(0.752) More Information | Similar Motifs Found | motif file (matrix) |
| 16 | C T A G C T G A G A C T G T A C C G T A C T G A C G T A T G A C G A C T C T A G G T A C G T C A G T C A | 1e-52 | -1.201e+02 | 2.39% | 0.12% | 102.6bp (155.2bp) | RUNX2/MA0511.2/Jaspar(0.685) More Information | Similar Motifs Found | motif file (matrix) |
| 17 | T G A C C G T A C T A G C T A G T C G A C A G T A T C G C G A T A C T G C T A G A T C G C G A T | 1e-51 | -1.195e+02 | 31.86% | 19.69% | 210.9bp (141.5bp) | ETS:RUNX(ETS,Runt)/Jurkat-RUNX1-ChIP-Seq(GSE17954)/Homer(0.802) More Information | Similar Motifs Found | motif file (matrix) |
| 18 | A G T C C G T A A C T G C G A T A C G T A G T C T C A G G T C A C T A G A G T C C G A T A C G T A G T C A G T C | 1e-51 | -1.181e+02 | 2.75% | 0.21% | 82.9bp (122.7bp) | POL008.1\_DCE\_S\_I/Jaspar(0.621) More Information | Similar Motifs Found | motif file (matrix) |
| 19 | G A C T A G C T C G A T A C T G G T C A C A G T A T G C C G A T C G A T G C T A C G A T T G A C | 1e-49 | -1.144e+02 | 11.47% | 4.50% | 144.4bp (128.9bp) | Mecom/MA0029.1/Jaspar(0.819) More Information | Similar Motifs Found | motif file (matrix) |
| 20 | A T G C G A T C G A C T C G T A G T C A A C G T A T G C G C T A T C G A C T A G G A T C G A T C G A C T | 1e-48 | -1.123e+02 | 3.02% | 0.30% | 98.8bp (147.8bp) | PH0026.1\_Duxbl/Jaspar(0.614) More Information | Similar Motifs Found | motif file (matrix) |
| 21 | C G T A A C T G G T C A G T A C C G T A C T A G A G T C C G T A A T C G G C A T | 1e-47 | -1.099e+02 | 45.95% | 32.71% | 187.2bp (143.0bp) | SCL(bHLH)/HPC7-Scl-ChIP-Seq(GSE13511)/Homer(0.742) More Information | Similar Motifs Found | motif file (matrix) |
| 22 | T C A G G C T A A G C T A T G C C G A T A G T C G C T A A C T G G C T A A G T C A C G T A C T G G A T C G A C T | 1e-45 | -1.055e+02 | 2.85% | 0.29% | 108.8bp (162.2bp) | Smad3(MAD)/NPC-Smad3-ChIP-Seq(GSE36673)/Homer(0.558) More Information | Similar Motifs Found | motif file (matrix) |
| 23 | C T A G A T G C A G T C A C G T A C G T A C T G A G T C C G T A A T C G C G A T A C G T A C G T | 1e-45 | -1.046e+02 | 2.09% | 0.11% | 95.6bp (143.9bp) | PB0146.1\_Mafk\_2/Jaspar(0.735) More Information | Similar Motifs Found | motif file (matrix) |
| 24 | C G T A A C T G T A C G G T A C C G T A A T C G A T C G A G T C A G T C A C G T A T G C A G T C A G C T C G A T | 1e-44 | -1.025e+02 | 2.16% | 0.13% | 90.3bp (157.1bp) | ZFX(Zf)/mES-Zfx-ChIP-Seq(GSE11431)/Homer(0.657) More Information | Similar Motifs Found | motif file (matrix) |
| 25 | A C T G A C T G A C T G G T C A A C G T A G T C A G T C C T A G A G T C A G T C A G T C G T C A | 1e-44 | -1.024e+02 | 2.49% | 0.20% | 106.8bp (123.9bp) | Sp5(Zf)/mES-Sp5.Flag-ChIP-Seq(GSE72989)/Homer(0.773) More Information | Similar Motifs Found | motif file (matrix) |
| 26 | A G T C A C G T A C T G A C G T A C T G A C G T A C G T A C G T C G T A A G T C | 1e-43 | -1.003e+02 | 3.55% | 0.54% | 94.9bp (146.7bp) | FOXP1/MA0481.1/Jaspar(0.869) More Information | Similar Motifs Found | motif file (matrix) |
| 27 | G C A T A T G C G A C T C A T G G T A C G C A T T C A G G C A T A C T G | 1e-43 | -9.997e+01 | 34.98% | 23.35% | 197.4bp (143.1bp) | Unknown-ESC-element(?)/mES-Nanog-ChIP-Seq(GSE11724)/Homer(0.748) More Information | Similar Motifs Found | motif file (matrix) |
| 28 | A G T C C T A G A G T C C G T A C T A G A G T C A C T G A G T C G T C A C G T A A C T G | 1e-41 | -9.670e+01 | 2.16% | 0.15% | 90.0bp (157.2bp) | POL009.1\_DCE\_S\_II/Jaspar(0.594) More Information | Similar Motifs Found | motif file (matrix) |
| 29 | C G T A A T C G C G T A C T A G G T C A C A T G C T A G A G T C C G T A C T A G A T C G T A G C C G T A | 1e-41 | -9.644e+01 | 2.65% | 0.28% | 122.3bp (147.7bp) | ZNF165(Zf)/WHIM12-ZNF165-ChIP-Seq(GSE65937)/Homer(0.577) More Information | Similar Motifs Found | motif file (matrix) |
| 30 | A G T C G T A C G T C A A G C T A C G T C T A G A G T C A G T C T A G C C G T A A C T G C T A G T A G C A C G T | 1e-41 | -9.636e+01 | 2.72% | 0.30% | 79.6bp (149.5bp) | ZNF416(Zf)/HEK293-ZNF416.GFP-ChIP-Seq(GSE58341)/Homer(0.644) More Information | Similar Motifs Found | motif file (matrix) |
| 31 | A C G T A C T G A G T C C G A T C G T A C A T G T A G C C G T A C T G A A G C T A T G C C G T A | 1e-41 | -9.560e+01 | 2.32% | 0.19% | 153.2bp (146.4bp) | Dux/MA0611.1/Jaspar(0.677) More Information | Similar Motifs Found | motif file (matrix) |
| 32 | A C T G A T C G C T A G A T C G A C T G G T C A A C T G A C G T | 1e-41 | -9.530e+01 | 28.25% | 17.82% | 210.1bp (130.9bp) | KLF16/MA0741.1/Jaspar(0.790) More Information | Similar Motifs Found | motif file (matrix) |
| 33 | A C G T A C T G A G T C A C G T C T A G A G C T C T A G A G T C A C G T C T G A A C T G T A G C G T C A | 1e-41 | -9.507e+01 | 2.32% | 0.19% | 103.3bp (169.4bp) | PB0207.1\_Zic3\_2/Jaspar(0.659) More Information | Similar Motifs Found | motif file (matrix) |
| 34 | A T C G G A C T C T A G A G T C G T C A A G T C A G T C A C G T A G T C A G T C A G T C A G T C A G T C C G T A | 1e-40 | -9.224e+01 | 2.12% | 0.16% | 156.2bp (139.2bp) | Zfp281(Zf)/ES-Zfp281-ChIP-Seq(GSE81042)/Homer(0.811) More Information | Similar Motifs Found | motif file (matrix) |
| 35 | C A T G T G A C C A G T A C T G A G C T A C G T G C T A C G T A T A G C G C T A | 1e-39 | -9.183e+01 | 42.61% | 30.70% | 182.6bp (140.0bp) | PB0109.1\_Bbx\_2/Jaspar(0.810) More Information | Similar Motifs Found | motif file (matrix) |
| 36 | C T A G A T G C C A G T G A C T C G A T A T G C A C G T | 1e-39 | -9.019e+01 | 59.28% | 46.91% | 184.4bp (138.9bp) | POL010.1\_DCE\_S\_III/Jaspar(0.665) More Information | Similar Motifs Found | motif file (matrix) |
| 37 | A C T G A G T C C G A T A C T G A G T C A G T C C A T G | 1e-38 | -8.856e+01 | 47.25% | 35.26% | 183.2bp (143.5bp) | POL010.1\_DCE\_S\_III/Jaspar(0.741) More Information | Similar Motifs Found | motif file (matrix) |
| 38 | A C G T A C T G A G T C A C G T A C T G A G T C A C T G A T C G A C G T C G T A A C T G | 1e-38 | -8.804e+01 | 2.49% | 0.27% | 107.3bp (165.2bp) | PB0207.1\_Zic3\_2/Jaspar(0.651) More Information | Similar Motifs Found | motif file (matrix) |
| 39 | A C G T A C T G A G T C A G C T C T A G A G T C A G T C A C G T A C G T A C G T A G T C C G A T A C T G A C G T | 1e-36 | -8.489e+01 | 2.82% | 0.40% | 111.4bp (151.1bp) | ETS:E-box(ETS,bHLH)/HPC7-Scl-ChIP-Seq(GSE22178)/Homer(0.567) More Information | Similar Motifs Found | motif file (matrix) |
| 40 | T A G C G T A C G A T C G T A C G A T C T G A C G A T C G T A C G T A C | 1e-35 | -8.215e+01 | 36.84% | 26.05% | 207.5bp (128.2bp) | Maz(Zf)/HepG2-Maz-ChIP-Seq(GSE31477)/Homer(0.885) More Information | Similar Motifs Found | motif file (matrix) |
| 41 | C G A T A C T G G A T C A G T C A G C T C G T A G A T C C G T A T C A G C G T A A C T G A T C G | 1e-35 | -8.213e+01 | 3.22% | 0.56% | 133.6bp (151.8bp) | TCFL2(HMG)/K562-TCF7L2-ChIP-Seq(GSE29196)/Homer(0.606) More Information | Similar Motifs Found | motif file (matrix) |
| 42 | T C A G A G C T A G C T G C A T C T A G C G A T T G A C C G T A A G C T | 1e-35 | -8.186e+01 | 37.60% | 26.76% | 187.3bp (136.6bp) | Tgif1(Homeobox)/mES-Tgif1-ChIP-Seq(GSE55404)/Homer(0.830) More Information | Similar Motifs Found | motif file (matrix) |
| 43 | A C T G T A C G A C G T A G T C A G T C G A C T C G T A G A T C C T A G A G T C A G T C A G T C C G T A T A G C | 1e-34 | -8.041e+01 | 1.86% | 0.14% | 138.7bp (154.8bp) | SP3/MA0746.1/Jaspar(0.710) More Information | Similar Motifs Found | motif file (matrix) |
| 44 | C T A G A T C G G A T C C T A G C A T G A T G C C G T A T C A G G A T C T A C G T C G A C T A G A T C G | 1e-34 | -7.900e+01 | 1.92% | 0.16% | 168.3bp (118.1bp) | Pax8(Paired,Homeobox)/Thyroid-Pax8-ChIP-Seq(GSE26938)/Homer(0.544) More Information | Similar Motifs Found | motif file (matrix) |
| 45 | A T C G A T G C T G A C A C G T A T G C A G T C A C T G A G C T C G T A A T C G | 1e-34 | -7.900e+01 | 2.55% | 0.35% | 164.4bp (148.9bp) | POL013.1\_MED-1/Jaspar(0.612) More Information | Similar Motifs Found | motif file (matrix) |
| 46 | C T G A T G A C C G T A A C G T A G T C G A C T C A T G | 1e-33 | -7.746e+01 | 34.42% | 24.18% | 175.7bp (140.0bp) | ZBTB18/MA0698.1/Jaspar(0.815) More Information | Similar Motifs Found | motif file (matrix) |
| 47 | A C G T G A T C A G T C A C T G A C G T A C T G A C T G A C T G A G T C C T A G A C G T C T G A A C T G | 1e-33 | -7.736e+01 | 1.56% | 0.08% | 116.5bp (138.3bp) | EGR3/MA0732.1/Jaspar(0.771) More Information | Similar Motifs Found | motif file (matrix) |
| 48 | G A C T C G A T T C A G A C G T A G T C C G T A A C T G G T C A | 1e-33 | -7.704e+01 | 47.61% | 36.42% | 181.8bp (137.5bp) | MEIS1/MA0498.2/Jaspar(0.740) More Information | Similar Motifs Found | motif file (matrix) |
| 49 | A C T G G A T C G C A T C A T G A C G T T A G C C G T A T A C G | 1e-32 | -7.569e+01 | 44.53% | 33.59% | 209.9bp (142.5bp) | Meis1(Homeobox)/MastCells-Meis1-ChIP-Seq(GSE48085)/Homer(0.829) More Information | Similar Motifs Found | motif file (matrix) |
| 50 | A C G T A C T G A G C T A C T G A G T C A T G C A G T C A C G T C T A G A G T C A G T C A G T C | 1e-31 | -7.299e+01 | 1.62% | 0.11% | 196.5bp (134.7bp) | ZNF467(Zf)/HEK293-ZNF467.GFP-ChIP-Seq(GSE58341)/Homer(0.662) More Information | Similar Motifs Found | motif file (matrix) |
| 51 | C T A G C T A G A G T C G T C A C T G A A C G T C A T G T A C G G A T C C A T G A C T G A C T G G A T C | 1e-31 | -7.266e+01 | 2.09% | 0.24% | 104.7bp (125.1bp) | YY2/MA0748.1/Jaspar(0.667) More Information | Similar Motifs Found | motif file (matrix) |
| 52 | C G T A A C T G G A C T A G T C A C G T C T A G A G T C | 1e-31 | -7.167e+01 | 32.19% | 22.57% | 185.3bp (139.4bp) | Smad3(MAD)/NPC-Smad3-ChIP-Seq(GSE36673)/Homer(0.789) More Information | Similar Motifs Found | motif file (matrix) |
| 53 | A G T C A G C T A G T C A C G T A C T G A G T C A C T G A C T G C G A T A C T G | 1e-30 | -6.955e+01 | 20.26% | 12.51% | 205.1bp (138.6bp) | Unknown-ESC-element(?)/mES-Nanog-ChIP-Seq(GSE11724)/Homer(0.669) More Information | Similar Motifs Found | motif file (matrix) |
| 54 | T G A C A C G T C A T G C G T A T C A G G T A C A C T G A T G C G A C T C A G T | 1e-29 | -6.697e+01 | 21.19% | 13.41% | 225.5bp (136.5bp) | Tcf7/MA0769.1/Jaspar(0.681) More Information | Similar Motifs Found | motif file (matrix) |
| 55 | C T A G T A G C G C A T A T C G A T G C A T G C A C G T G C A T G A C T | 1e-28 | -6.536e+01 | 22.31% | 14.43% | 193.7bp (138.8bp) | Nr2e1/MA0676.1/Jaspar(0.612) More Information | Similar Motifs Found | motif file (matrix) |
| 56 | A G T C A C T G G A C T A G C T A C T G C G A T C G A T A C G T C G T A | 1e-27 | -6.258e+01 | 3.51% | 0.90% | 109.6bp (141.6bp) | Foxj2/MA0614.1/Jaspar(0.773) More Information | Similar Motifs Found | motif file (matrix) |
| 57 | G C A T C A G T G C T A C G T A A C T G C G A T T A G C G A C T C A T G A G T C C G T A A C T G G T C A C T A G | 1e-26 | -6.197e+01 | 1.92% | 0.25% | 128.4bp (145.0bp) | MafA(bZIP)/Islet-MafA-ChIP-Seq(GSE30298)/Homer(0.644) More Information | Similar Motifs Found | motif file (matrix) |
| 58 | T A C G A T G C G C T A C T A G A T C G G T A C C G A T A T C G T G A C G A T C G C A T | 1e-26 | -6.171e+01 | 30.74% | 21.94% | 192.2bp (141.1bp) | Tlx?(NR)/NPC-H3K4me1-ChIP-Seq(GSE16256)/Homer(0.690) More Information | Similar Motifs Found | motif file (matrix) |
| 59 | G T A C G A T C C T A G G A T C G T A C C A G T A C G T A T C G A T G C C G T A | 1e-25 | -5.952e+01 | 3.38% | 0.88% | 104.4bp (135.8bp) | INSM1/MA0155.1/Jaspar(0.618) More Information | Similar Motifs Found | motif file (matrix) |
| 60 | G A T C G C A T C G T A T A C G A G T C C T G A T C A G T C A G A T G C | 1e-25 | -5.914e+01 | 19.36% | 12.33% | 186.3bp (140.0bp) | Unknown-ESC-element(?)/mES-Nanog-ChIP-Seq(GSE11724)/Homer(0.626) More Information | Similar Motifs Found | motif file (matrix) |
| 61 | C G T A A G T C A G T C A G T C C A G T G T A C A G T C A C T G C G T A A C T G A G T C G A T C C G T A | 1e-25 | -5.882e+01 | 1.36% | 0.10% | 124.7bp (145.9bp) | Reverb(NR),DR2/RAW-Reverba.biotin-ChIP-Seq(GSE45914)/Homer(0.561) More Information | Similar Motifs Found | motif file (matrix) |
| 62 | A T C G A T C G G C T A A C T G A C T G C T G A A C T G A T G C A T G C | 1e-25 | -5.811e+01 | 13.13% | 7.41% | 231.8bp (135.1bp) | Znf263(Zf)/K562-Znf263-ChIP-Seq(GSE31477)/Homer(0.712) More Information | Similar Motifs Found | motif file (matrix) |
| 63 | C T A G A G T C C T A G C A T G A T C G C T G A A C G T G C T A C G A T T G C A C T G A A C G T A G T C C G A T | 1e-25 | -5.760e+01 | 1.39% | 0.12% | 131.9bp (164.4bp) | GATA(Zf),IR3/iTreg-Gata3-ChIP-Seq(GSE20898)/Homer(0.642) More Information | Similar Motifs Found | motif file (matrix) |
| 64 | C G A T A C G T A C G T A T G C G A C T T A G C C G T A A C T G G C T A T G C A | 1e-24 | -5.605e+01 | 5.17% | 1.92% | 150.0bp (127.1bp) | STAT5(Stat)/mCD4+-Stat5-ChIP-Seq(GSE12346)/Homer(0.934) More Information | Similar Motifs Found | motif file (matrix) |
| 65 | A C G T C G T A T C A G A C T G C G T A A G T C A G T C A G T C A C G T G T A C | 1e-24 | -5.535e+01 | 1.62% | 0.19% | 152.2bp (138.1bp) | LRF(Zf)/Erythroblasts-ZBTB7A-ChIP-Seq(GSE74977)/Homer(0.735) More Information | Similar Motifs Found | motif file (matrix) |
| 66 | A C G T A T C G C G T A A C G T C G T A C G T A G C T A | 1e-23 | -5.417e+01 | 18.53% | 11.92% | 159.6bp (132.6bp) | GATA5/MA0766.1/Jaspar(0.822) More Information | Similar Motifs Found | motif file (matrix) |
| 67 | C G A T C G T A A G T C A C G T T C A G A G T C A G C T T C G A A G C T G A T C A G C T A C G T | 1e-23 | -5.385e+01 | 2.12% | 0.38% | 137.1bp (130.8bp) | PB0126.1\_Gata5\_2/Jaspar(0.638) More Information | Similar Motifs Found | motif file (matrix) |
| 68 | A C G T G C A T A C T G A C G T C T G A C T A G A G C T A G T C A C G T G T C A | 1e-21 | -4.994e+01 | 11.77% | 6.73% | 180.7bp (132.5bp) | PB0196.1\_Zbtb7b\_2/Jaspar(0.634) More Information | Similar Motifs Found | motif file (matrix) |
| 69 | A G T C C G A T C T A G C T A G C G T A C T G A A C T G A T C G A C G T C G T A C G T A T C G A C G A T A C T G | 1e-21 | -4.896e+01 | 0.90% | 0.04% | 120.7bp (133.2bp) | Barhl1/MA0877.1/Jaspar(0.638) More Information | Similar Motifs Found | motif file (matrix) |
| 70 | C G T A T G A C C G T A C T A G C A T G G T C A G C T A C T A G T G A C C G A T A T C G A G C T A C G T C G A T | 1e-20 | -4.668e+01 | 2.95% | 0.84% | 135.4bp (127.7bp) | PB0047.1\_Myf6\_1/Jaspar(0.622) More Information | Similar Motifs Found | motif file (matrix) |
| 71 | A T C G C G T A A T C G A C T G A G T C A G T C A C G T | 1e-20 | -4.640e+01 | 19.66% | 13.33% | 222.5bp (136.7bp) | ZFX(Zf)/mES-Zfx-ChIP-Seq(GSE11431)/Homer(0.919) More Information | Similar Motifs Found | motif file (matrix) |
| 72 | A C G T A C G T A C T G A C T G A C G T C G A T A G C T A C T G A C G T A G T C C G A T | 1e-19 | -4.522e+01 | 1.76% | 0.31% | 104.0bp (128.0bp) | PB0166.1\_Sox12\_2/Jaspar(0.634) More Information | Similar Motifs Found | motif file (matrix) |
| 73 | C G T A C G T A A C G T A G T C C G T A A C T G A G T C A C T G C G T A A C T G C T G A | 1e-17 | -4.009e+01 | 1.03% | 0.10% | 109.6bp (131.5bp) | PB0139.1\_Irf5\_2/Jaspar(0.662) More Information | Similar Motifs Found | motif file (matrix) |
| 74 | A T G C A C G T A C G T A G T C C G T A A T C G C G T A | 1e-17 | -3.959e+01 | 31.50% | 24.37% | 178.2bp (140.4bp) | POL008.1\_DCE\_S\_I/Jaspar(0.709) More Information | Similar Motifs Found | motif file (matrix) |
| 75 | T A G C A G T C C T A G A C T G A G C T C G A T A G T C A C T G | 1e-15 | -3.539e+01 | 1.82% | 0.44% | 123.2bp (133.6bp) | AMYB(HTH)/Testes-AMYB-ChIP-Seq(GSE44588)/Homer(0.636) More Information | Similar Motifs Found | motif file (matrix) |
| 76 | A T C G A C G T C A T G G T A C C G T A A G T C C G A T A T C G A C G T A T C G | 1e-15 | -3.531e+01 | 16.78% | 11.63% | 186.1bp (145.9bp) | HNF4a(NR),DR1/HepG2-HNF4a-ChIP-Seq(GSE25021)/Homer(0.669) More Information | Similar Motifs Found | motif file (matrix) |
| 77 | C G T A C G T A C G T A G T A C A T C G A C T G A G T C T C G A A G T C C G T A A G T C A G T C C G T A | 1e-15 | -3.488e+01 | 0.93% | 0.10% | 144.7bp (156.9bp) | PB0208.1\_Zscan4\_2/Jaspar(0.630) More Information | Similar Motifs Found | motif file (matrix) |
| 78 | A T C G C G T A A C G T A C G T T G C A G C A T T G C A C G A T G T A C | 1e-15 | -3.488e+01 | 7.92% | 4.47% | 181.8bp (130.3bp) | GATA(Zf),IR3/iTreg-Gata3-ChIP-Seq(GSE20898)/Homer(0.786) More Information | Similar Motifs Found | motif file (matrix) |
| 79 | A C G T A G T C A G T C A C G T C G T A A G T C A C T G A G T C | 1e-15 | -3.456e+01 | 1.16% | 0.18% | 155.0bp (139.8bp) | PB0114.1\_Egr1\_2/Jaspar(0.682) More Information | Similar Motifs Found | motif file (matrix) |
| 80 | C G T A C G T A A T C G A C T G G T C A A C T G A C T G A G T C | 1e-14 | -3.336e+01 | 6.43% | 3.42% | 156.4bp (131.0bp) | PB0076.1\_Sp4\_1/Jaspar(0.676) More Information | Similar Motifs Found | motif file (matrix) |
| 81 | C G T A A G T C C G T A A C G T A G T C C G T A A G T C C G T A A C T G | 1e-13 | -3.158e+01 | 2.22% | 0.70% | 140.0bp (136.4bp) | Tcf4(HMG)/Hct116-Tcf4-ChIP-Seq(SRA012054)/Homer(0.764) More Information | Similar Motifs Found | motif file (matrix) |
