# Supplementary material for: Mechanisms governing the pioneering and redistribution capabilities of the non-classical pioneer PU.1
Source: Nat Commun. 2020 Jan 21;11:402. doi: 10.1038/s41467-019-13960-2 (PMC6972792; doi:10.1038/s41467-019-13960-2)
Supplement: Supplementary file 7 — Source data [file 41467_2019_13960_MOESM7_ESM.zip › Source_Data/Figure5/Figure5A_MotifScanOutput/homerResults/motif48.similar.html]

motif48

## Information for motif48

G
A
C
T
C
G
A
T
T
C
A
G
A
C
G
T
A
G
T
C
C
G
T
A
A
C
T
G
G
T
C
A
  
Reverse Opposite:  

C
A
G
T
T
G
A
C
C
G
A
T
T
C
A
G
T
G
C
A
A
G
T
C
G
C
T
A
C
T
G
A
  

|  |  |
| --- | --- |
| p-value: | 1e-33 |
| log p-value: | -7.704e+01 |
| Information Content per bp: | 1.616 |
| Number of Target Sequences with motif | 1436.0 |
| Percentage of Target Sequences with motif | 47.61% |
| Number of Background Sequences with motif | 16613.9 |
| Percentage of Background Sequences with motif | 36.42% |
| Average Position of motif in Targets | 240.6 +/- 181.8bp |
| Average Position of motif in Background | 209.0 +/- 137.5bp |
| Strand Bias (log2 ratio + to - strand density) | 0.0 |
| Multiplicity (# of sites on avg that occur together) | 1.37 |
| Motif File: | file (matrix) reverse opposite |

### Similar de novo motifs found

|  |  |  |  |  |  |  |  |
| --- | --- | --- | --- | --- | --- | --- | --- |
| Rank | Match Score | Redundant Motif | P-value | log P-value | % of Targets | % of Background | Motif file |
| 1 | 0.834 | A C G T A G T C A C G T A C T G G T C A A T C G G T C A | 1e-32 | -74.118755 | 33.02% | 23.15% | motif file (matrix) |
| 2 | 0.751 | G C A T A C G T T G A C G C A T A C T G G T C A A T C G G T C A C T G A | 1e-18 | -43.051487 | 6.17% | 2.89% | motif file (matrix) |
