# Supplementary material for: Mechanisms governing the pioneering and redistribution capabilities of the non-classical pioneer PU.1
Source: Nat Commun. 2020 Jan 21;11:402. doi: 10.1038/s41467-019-13960-2 (PMC6972792; doi:10.1038/s41467-019-13960-2)
Supplement: Supplementary file 7 — Source data [file 41467_2019_13960_MOESM7_ESM.zip › Source_Data/Figure5/Figure5A_MotifScanOutput/homerResults/motif1.similar.html]

motif1

## Information for motif1

C
A
G
T
A
C
G
T
G
A
C
T
C
A
G
T
A
C
T
G
G
C
A
T
C
A
T
G
A
T
C
G
A
G
C
T
G
A
C
T
C
G
A
T
  
Reverse Opposite:  

G
C
T
A
C
T
G
A
T
C
G
A
T
A
G
C
G
T
A
C
C
G
T
A
T
G
A
C
G
T
C
A
C
T
G
A
T
G
C
A
G
T
C
A
  

|  |  |
| --- | --- |
| p-value: | 1e-455 |
| log p-value: | -1.049e+03 |
| Information Content per bp: | 1.653 |
| Number of Target Sequences with motif | 1580.0 |
| Percentage of Target Sequences with motif | 52.39% |
| Number of Background Sequences with motif | 6841.2 |
| Percentage of Background Sequences with motif | 15.00% |
| Average Position of motif in Targets | 225.9 +/- 148.6bp |
| Average Position of motif in Background | 203.6 +/- 138.1bp |
| Strand Bias (log2 ratio + to - strand density) | 0.1 |
| Multiplicity (# of sites on avg that occur together) | 1.34 |
| Motif File: | file (matrix) reverse opposite |

### Similar de novo motifs found

|  |  |  |  |  |  |  |  |
| --- | --- | --- | --- | --- | --- | --- | --- |
| Rank | Match Score | Redundant Motif | P-value | log P-value | % of Targets | % of Background | Motif file |
| 1 | 0.989 | G A C T C A G T C A T G G C A T A T C G C T A G A G C T A C G T C G A T | 1e-434 | -1001.142377 | 53.45% | 16.33% | motif file (matrix) |
| 2 | 0.983 | T G A C G C T A T C G A T G C A A G T C A G T C C G T A G T A C G C T A C T G A | 1e-418 | -963.804800 | 70.23% | 30.05% | motif file (matrix) |
| 3 | 0.968 | C A G T C T A G G A C T T C A G A T C G A G C T G A C T C A G T | 1e-411 | -947.301466 | 52.29% | 16.30% | motif file (matrix) |
| 4 | 0.969 | A G C T C A G T G C A T C A G T A C G T G A C T C G A T A C T G C G A T A C T G A C T G A G C T G A C T | 1e-402 | -926.778251 | 63.86% | 25.24% | motif file (matrix) |
| 5 | 0.964 | A C G T A C T G A G C T A C T G A C T G A C G T A C G T | 1e-351 | -810.051718 | 36.47% | 8.59% | motif file (matrix) |
| 6 | 0.912 | A G C T C A G T C A G T C A G T A C T G G C A T C G A T A C T G C G A T A C T G A C T G A G C T | 1e-315 | -725.494921 | 52.16% | 19.74% | motif file (matrix) |
| 7 | 0.969 | G C T A C T G A T C G A A T G C A G T C C T G A G A T C C G T A T C G A | 1e-303 | -698.627914 | 74.73% | 40.20% | motif file (matrix) |
| 8 | 0.851 | G C T A C T G A A G T C T A G C C G T A G T A C C T G A C T G A | 1e-247 | -569.166634 | 27.72% | 6.73% | motif file (matrix) |
| 9 | 0.889 | C T A G T A G C G C A T A T G C C G A T A C T G C A G T C T A G T A C G A C G T A G C T C A G T | 1e-199 | -460.492560 | 42.44% | 17.78% | motif file (matrix) |
| 10 | 0.830 | A T G C A T G C C T G A A G T C C G T A C T A G T G C A | 1e-173 | -398.592797 | 67.27% | 41.05% | motif file (matrix) |
| 11 | 0.861 | A G C T C G A T T G C A A T G C T G A C C G T A T A G C C G T A | 1e-172 | -397.936544 | 63.26% | 37.13% | motif file (matrix) |
| 12 | 0.804 | G T C A T C G A T G C A A G T C G T A C G C T A A G T C G C T A A C G T G A T C G A T C G C A T A T C G G A C T | 1e-133 | -307.474954 | 26.49% | 9.97% | motif file (matrix) |
| 13 | 0.809 | A G T C G C A T A G C T C A G T A T C G G C T A C G A T A C T G C G A T C A T G C A T G A G C T A C G T C A G T | 1e-126 | -291.170278 | 18.37% | 5.37% | motif file (matrix) |
| 14 | 0.790 | C G A T A C T G C A G T A C T G A T C G A G C T A C G T A C G T A C T G A C G T A C T G A C T G A C G T A C G T | 1e-106 | -244.392794 | 37.90% | 19.90% | motif file (matrix) |
| 15 | 0.786 | C G T A C G T A A G T C A G T C A C G T A G T C C G T A | 1e-15 | -34.889292 | 11.67% | 7.41% | motif file (matrix) |
