# Supplementary material for: Mechanisms governing the pioneering and redistribution capabilities of the non-classical pioneer PU.1
Source: Nat Commun. 2020 Jan 21;11:402. doi: 10.1038/s41467-019-13960-2 (PMC6972792; doi:10.1038/s41467-019-13960-2)
Supplement: Supplementary file 7 — Source data [file 41467_2019_13960_MOESM7_ESM.zip › Source_Data/Figure5/Figure5A_MotifScanOutput/homerResults/motif10.similar.html]

motif10

## Information for motif10

G
T
C
A
T
C
G
A
A
G
T
C
A
G
T
C
A
G
T
C
G
T
C
A
A
G
T
C
C
T
A
G
  
Reverse Opposite:  

A
G
T
C
A
C
T
G
A
C
G
T
A
C
T
G
A
C
T
G
A
C
T
G
A
G
C
T
A
C
G
T
  

|  |  |
| --- | --- |
| p-value: | 1e-59 |
| log p-value: | -1.361e+02 |
| Information Content per bp: | 1.818 |
| Number of Target Sequences with motif | 734.0 |
| Percentage of Target Sequences with motif | 24.34% |
| Number of Background Sequences with motif | 5905.7 |
| Percentage of Background Sequences with motif | 12.95% |
| Average Position of motif in Targets | 258.9 +/- 181.0bp |
| Average Position of motif in Background | 212.5 +/- 140.8bp |
| Strand Bias (log2 ratio + to - strand density) | -0.1 |
| Multiplicity (# of sites on avg that occur together) | 1.16 |
| Motif File: | file (matrix) reverse opposite |

### Similar de novo motifs found

|  |  |  |  |  |  |  |  |
| --- | --- | --- | --- | --- | --- | --- | --- |
| Rank | Match Score | Redundant Motif | P-value | log P-value | % of Targets | % of Background | Motif file |
| 1 | 0.765 | A T G C A G T C A G T C G T C A A G T C C T G A A G T C | 1e-31 | -73.269137 | 41.61% | 31.03% | motif file (matrix) |
| 2 | 0.764 | A G C T G A T C A G T C A C T G A C G T A C T G A C T G A C T G A G T C C T A G | 1e-23 | -55.127823 | 2.42% | 0.49% | motif file (matrix) |
