# Supplementary material for: Mechanisms governing the pioneering and redistribution capabilities of the non-classical pioneer PU.1
Source: Nat Commun. 2020 Jan 21;11:402. doi: 10.1038/s41467-019-13960-2 (PMC6972792; doi:10.1038/s41467-019-13960-2)
Supplement: Supplementary file 7 — Source data [file 41467_2019_13960_MOESM7_ESM.zip › Source_Data/Figure5/Figure5A_MotifScanOutput/homerResults/motif21.similar.html]

motif21

## Information for motif21

C
G
T
A
A
C
T
G
G
T
C
A
G
T
A
C
C
G
T
A
C
T
A
G
A
G
T
C
C
G
T
A
A
T
C
G
G
C
A
T
  
Reverse Opposite:  

C
G
T
A
T
A
G
C
G
C
A
T
T
C
A
G
G
A
T
C
G
C
A
T
C
A
T
G
C
A
G
T
T
G
A
C
G
C
A
T
  

|  |  |
| --- | --- |
| p-value: | 1e-47 |
| log p-value: | -1.099e+02 |
| Information Content per bp: | 1.536 |
| Number of Target Sequences with motif | 1386.0 |
| Percentage of Target Sequences with motif | 45.95% |
| Number of Background Sequences with motif | 14919.3 |
| Percentage of Background Sequences with motif | 32.71% |
| Average Position of motif in Targets | 236.7 +/- 187.2bp |
| Average Position of motif in Background | 208.4 +/- 143.0bp |
| Strand Bias (log2 ratio + to - strand density) | 0.0 |
| Multiplicity (# of sites on avg that occur together) | 1.43 |
| Motif File: | file (matrix) reverse opposite |

### Similar de novo motifs found

|  |  |  |  |  |  |  |  |
| --- | --- | --- | --- | --- | --- | --- | --- |
| Rank | Match Score | Redundant Motif | P-value | log P-value | % of Targets | % of Background | Motif file |
| 1 | 0.755 | A C G T C G T A A G T C A C G T A C T G A G T C A C G T A C T G A G C T A G T C A C G T A G C T A G C T | 1e-29 | -68.250689 | 1.56% | 0.12% | motif file (matrix) |
