# Supplementary material for: Mechanisms governing the pioneering and redistribution capabilities of the non-classical pioneer PU.1
Source: Nat Commun. 2020 Jan 21;11:402. doi: 10.1038/s41467-019-13960-2 (PMC6972792; doi:10.1038/s41467-019-13960-2)
Supplement: Supplementary file 7 — Source data [file 41467_2019_13960_MOESM7_ESM.zip › Source_Data/Figure5/Figure5A_MotifScanOutput/homerResults/motif51.similar.html]

motif51

## Information for motif51

C
T
A
G
C
T
A
G
A
G
T
C
G
T
C
A
C
T
G
A
A
C
G
T
C
A
T
G
T
A
C
G
G
A
T
C
C
A
T
G
A
C
T
G
A
C
T
G
G
A
T
C
  
Reverse Opposite:  

C
T
A
G
A
G
T
C
A
G
T
C
G
T
A
C
C
T
A
G
A
T
G
C
G
T
A
C
G
T
C
A
A
G
C
T
C
A
G
T
C
T
A
G
A
G
T
C
A
G
T
C
  

|  |  |
| --- | --- |
| p-value: | 1e-31 |
| log p-value: | -7.266e+01 |
| Information Content per bp: | 1.771 |
| Number of Target Sequences with motif | 63.0 |
| Percentage of Target Sequences with motif | 2.09% |
| Number of Background Sequences with motif | 108.7 |
| Percentage of Background Sequences with motif | 0.24% |
| Average Position of motif in Targets | 165.6 +/- 104.7bp |
| Average Position of motif in Background | 188.5 +/- 125.1bp |
| Strand Bias (log2 ratio + to - strand density) | 0.1 |
| Multiplicity (# of sites on avg that occur together) | 1.00 |
| Motif File: | file (matrix) reverse opposite |

### Similar de novo motifs found

|  |  |  |  |  |  |  |  |
| --- | --- | --- | --- | --- | --- | --- | --- |
| Rank | Match Score | Redundant Motif | P-value | log P-value | % of Targets | % of Background | Motif file |
| 1 | 0.863 | A G T C C G T A C G T A A C G T A C T G A C T G A G T C C T A G C T A G A C T G G A T C C A T G | 1e-31 | -72.369659 | 1.53% | 0.09% | motif file (matrix) |
| 2 | 0.879 | C T G A A C T G A G T C C G T A T G C A A C G T A C T G A C T G G A T C C T A G A C T G | 1e-28 | -66.608883 | 2.35% | 0.36% | motif file (matrix) |
| 3 | 0.797 | A G T C C G T A G T C A A C G T A C T G A C T G A G T C C T A G A C T G | 1e-19 | -45.233507 | 3.48% | 1.15% | motif file (matrix) |
