# Supplementary material for: Mechanisms governing the pioneering and redistribution capabilities of the non-classical pioneer PU.1
Source: Nat Commun. 2020 Jan 21;11:402. doi: 10.1038/s41467-019-13960-2 (PMC6972792; doi:10.1038/s41467-019-13960-2)
Supplement: Supplementary file 7 — Source data [file 41467_2019_13960_MOESM7_ESM.zip › Source_Data/Figure5/Figure5A_MotifScanOutput/homerResults/motif32.similar.html]

motif32

## Information for motif32

A
C
T
G
A
T
C
G
C
T
A
G
A
T
C
G
A
C
T
G
G
T
C
A
A
C
T
G
A
C
G
T
  
Reverse Opposite:  

T
G
C
A
A
G
T
C
C
A
G
T
A
G
T
C
A
T
G
C
A
G
T
C
T
A
G
C
A
G
T
C
  

|  |  |
| --- | --- |
| p-value: | 1e-41 |
| log p-value: | -9.530e+01 |
| Information Content per bp: | 1.869 |
| Number of Target Sequences with motif | 852.0 |
| Percentage of Target Sequences with motif | 28.25% |
| Number of Background Sequences with motif | 8128.5 |
| Percentage of Background Sequences with motif | 17.82% |
| Average Position of motif in Targets | 256.0 +/- 210.1bp |
| Average Position of motif in Background | 208.9 +/- 130.9bp |
| Strand Bias (log2 ratio + to - strand density) | 0.0 |
| Multiplicity (# of sites on avg that occur together) | 1.19 |
| Motif File: | file (matrix) reverse opposite |

### Similar de novo motifs found

|  |  |  |  |  |  |  |  |
| --- | --- | --- | --- | --- | --- | --- | --- |
| Rank | Match Score | Redundant Motif | P-value | log P-value | % of Targets | % of Background | Motif file |
| 1 | 0.807 | A G T C A G T C A G T C A G T C A G T C T G A C A G T C | 1e-37 | -86.059283 | 16.84% | 9.10% | motif file (matrix) |
| 2 | 0.923 | C G T A G T A C A C G T A G T C A G T C A G T C G T A C A G T C A T C G | 1e-25 | -58.193728 | 16.35% | 9.93% | motif file (matrix) |
| 3 | 0.854 | A C T G A C T G C G T A A C T G A C T G C G T A A C T G | 1e-15 | -35.722131 | 17.47% | 12.19% | motif file (matrix) |
