# Supplementary material for: Mechanisms governing the pioneering and redistribution capabilities of the non-classical pioneer PU.1
Source: Nat Commun. 2020 Jan 21;11:402. doi: 10.1038/s41467-019-13960-2 (PMC6972792; doi:10.1038/s41467-019-13960-2)
Supplement: Supplementary file 7 — Source data [file 41467_2019_13960_MOESM7_ESM.zip › Source_Data/Figure5/Figure5A_MotifScanOutput/homerResults/motif35.similar.html]

motif35

## Information for motif35

C
A
T
G
T
G
A
C
C
A
G
T
A
C
T
G
A
G
C
T
A
C
G
T
G
C
T
A
C
G
T
A
T
A
G
C
G
C
T
A
  
Reverse Opposite:  

C
G
A
T
A
T
C
G
G
C
A
T
C
A
G
T
G
T
C
A
T
C
G
A
T
G
A
C
G
T
C
A
A
C
T
G
G
T
A
C
  

|  |  |
| --- | --- |
| p-value: | 1e-39 |
| log p-value: | -9.183e+01 |
| Information Content per bp: | 1.532 |
| Number of Target Sequences with motif | 1285.0 |
| Percentage of Target Sequences with motif | 42.61% |
| Number of Background Sequences with motif | 14006.3 |
| Percentage of Background Sequences with motif | 30.70% |
| Average Position of motif in Targets | 241.7 +/- 182.6bp |
| Average Position of motif in Background | 208.5 +/- 140.0bp |
| Strand Bias (log2 ratio + to - strand density) | 0.1 |
| Multiplicity (# of sites on avg that occur together) | 1.27 |
| Motif File: | file (matrix) reverse opposite |

### Similar de novo motifs found

|  |  |  |  |  |  |  |  |
| --- | --- | --- | --- | --- | --- | --- | --- |
| Rank | Match Score | Redundant Motif | P-value | log P-value | % of Targets | % of Background | Motif file |
| 1 | 0.762 | C G A T C T G A C G T A T G A C G C T A A C T G A G T C | 1e-34 | -79.065638 | 48.97% | 37.58% | motif file (matrix) |
| 2 | 0.890 | T C A G T G A C C G A T A C T G C G A T C A G T G C T A G T C A | 1e-32 | -74.270270 | 55.80% | 44.61% | motif file (matrix) |
