# Supplementary material for: Mechanisms governing the pioneering and redistribution capabilities of the non-classical pioneer PU.1
Source: Nat Commun. 2020 Jan 21;11:402. doi: 10.1038/s41467-019-13960-2 (PMC6972792; doi:10.1038/s41467-019-13960-2)
Supplement: Supplementary file 7 — Source data [file 41467_2019_13960_MOESM7_ESM.zip › Source_Data/Figure5/Figure5A_MotifScanOutput/homerResults/motif20.similar.html]

motif20

## Information for motif20

A
T
G
C
G
A
T
C
G
A
C
T
C
G
T
A
G
T
C
A
A
C
G
T
A
T
G
C
G
C
T
A
T
C
G
A
C
T
A
G
G
A
T
C
G
A
T
C
G
A
C
T
  
Reverse Opposite:  

C
T
G
A
C
T
A
G
C
T
A
G
A
G
T
C
A
C
G
T
C
G
A
T
T
A
C
G
T
G
C
A
C
A
G
T
G
C
A
T
C
T
G
A
C
A
T
G
T
A
C
G
  

|  |  |
| --- | --- |
| p-value: | 1e-48 |
| log p-value: | -1.123e+02 |
| Information Content per bp: | 1.758 |
| Number of Target Sequences with motif | 91.0 |
| Percentage of Target Sequences with motif | 3.02% |
| Number of Background Sequences with motif | 137.9 |
| Percentage of Background Sequences with motif | 0.30% |
| Average Position of motif in Targets | 172.5 +/- 98.8bp |
| Average Position of motif in Background | 234.3 +/- 147.8bp |
| Strand Bias (log2 ratio + to - strand density) | 0.2 |
| Multiplicity (# of sites on avg that occur together) | 1.00 |
| Motif File: | file (matrix) reverse opposite |

### Similar de novo motifs found

|  |  |  |  |  |  |  |  |
| --- | --- | --- | --- | --- | --- | --- | --- |
| Rank | Match Score | Redundant Motif | P-value | log P-value | % of Targets | % of Background | Motif file |
| 1 | 0.890 | A G T C A G C T C G T A G C A T A C T G A T G C G T C A C G T A A C T G A T G C A G T C A G C T | 1e-44 | -101.812847 | 2.65% | 0.25% | motif file (matrix) |
| 2 | 0.904 | G A T C A C G T C G T A G T C A A C T G A G T C G C T A C T G A A C T G A G T C A G T C | 1e-42 | -96.988461 | 2.92% | 0.35% | motif file (matrix) |
| 3 | 0.826 | A G T C A C G T C G T A G T C A A C T G A G T C C T G A C G T A A C T G A G T C | 1e-34 | -80.540199 | 2.82% | 0.43% | motif file (matrix) |
| 4 | 0.803 | A C T G A G T C A C G T A C G T A C T G G T A C A C G T C G A T C G T A | 1e-29 | -68.674240 | 3.95% | 1.03% | motif file (matrix) |
| 5 | 0.763 | A G C T C T G A G T A C G A C T G A T C G T C A C T G A T C A G T A G C A G T C G C A T A T G C G T C A | 1e-13 | -31.367448 | 2.92% | 1.10% | motif file (matrix) |
